# Effects of Management on Lichen Species Richness, Ecological Traits and Community Structure in the Rodnei Mountains National Park (Romania)

**DOI:** 10.1371/journal.pone.0145808

**Published:** 2015-12-30

**Authors:** Ioana Violeta Ardelean, Christine Keller, Christoph Scheidegger

**Affiliations:** 1 Swiss Federal Research Institute WSL, 8903 Birmensdorf, Switzerland; 2 National Institute of Research and Development for Biological Sciences, Cluj–Napoca, Romania; National Taiwan University, TAIWAN

## Abstract

Lichens are valuable bio-indicators for evaluating the consequences of human activities that are increasingly changing the earth’s ecosystems. Since a major objective of national parks is the preservation of biodiversity, our aim is to analyse how natural resource management, the availability of lichen substrates and environmental parameters influence lichen diversity in Rodnei Mountains National Park situated in the Eastern Carpathians. Three main types of managed vegetation were investigated: the transhumance systems in alpine meadows, timber exploitation in mixed and pure spruce forests, and the corresponding conserved sites. The data were sampled following a replicated design. For the analysis, we considered not only all lichen species, but also species groups from different substrates such as soil, trees and deadwood. The lichen diversity was described according to species richness, red-list status and substrate-specialist species richness. The variation in species composition was related to the environmental variables. Habitat management was found to negatively influence species richness and alter the lichen community composition, particularly for threatened and substrate-specialist species. It reduced the mean level of threatened species richness by 59%, when all lichen species were considered, and by 81%, when only epiphytic lichens were considered. Management-induced disturbance significantly decreased lichen species richness in forest landscapes with long stand continuity. The diversity patterns of the lichens indicate a loss of species richness and change in species composition in areas where natural resources are still exploited inside the borders of the national park. It is thus imperative for protected areas, in particular old-growth forests and alpine meadows, to receive more protection than they have received in the past to ensure populations of the characteristic species remain viable in the future.

## Introduction

The loss of taxonomic, phylogenetic, genetic, and functional diversity in ecosystems worldwide is currently taking place very rapidly due to human activities [1]. To slow down the rate of loss, effective protection measures are essential. Today more than 100 000 sites are protected, covering over 12.7% of the world’s terrestrial area according to the World Database on Protected Areas (WDPA). The size of these areas provides, however, little information on how effective they are for biodiversity conservation [2]. For example, while in Europe only very small surfaces of intact old-growth forests (0.2% of the total forest area) are still present [3], in Romania the loss of old-growth forest area is an on-going process. Moreover, 72% of the exploitations of old-growth forests are taking place in protected areas [4].

Lichens are well known bio-indicators and have been frequently used to infer habitat continuity [5, 6]. Studies carried out in habitats with increasing anthropogenic pressure show that lichens have more differentiated patterns of species diversity related to habitat change than other groups of organisms in the same sites [7, 8]. Lichen communities differ greatly in natural and secondary forests in terms of both species richness and composition [9]. Among lichens, the red-listed species are the most threatened by habitat management, and their decline often indicates substantial changes in lichen diversity and composition generally [10].

Functional traits have rarely been used to characterize the responses of lichens to habitat management or other environmental variables. However, the species richness of the functional groups, i.e. their rarity and substrate specialization in relation to total species richness, have been analysed and new patterns determined [11, 12]. The differences found between the lichen communities in conserved and managed habitats are often connected to substrate preferences and the distribution area of the species [12, 13]. By comparing the responses of lichen communities from different substrate types, complex diversity patterns could be derived. The main substrate types include living trees, deadwood, soil and rocks, which host different lichen communities [14]. Nevertheless, few studies of lichen diversity have yet considered multiple substrate types.

**Fig 1:**
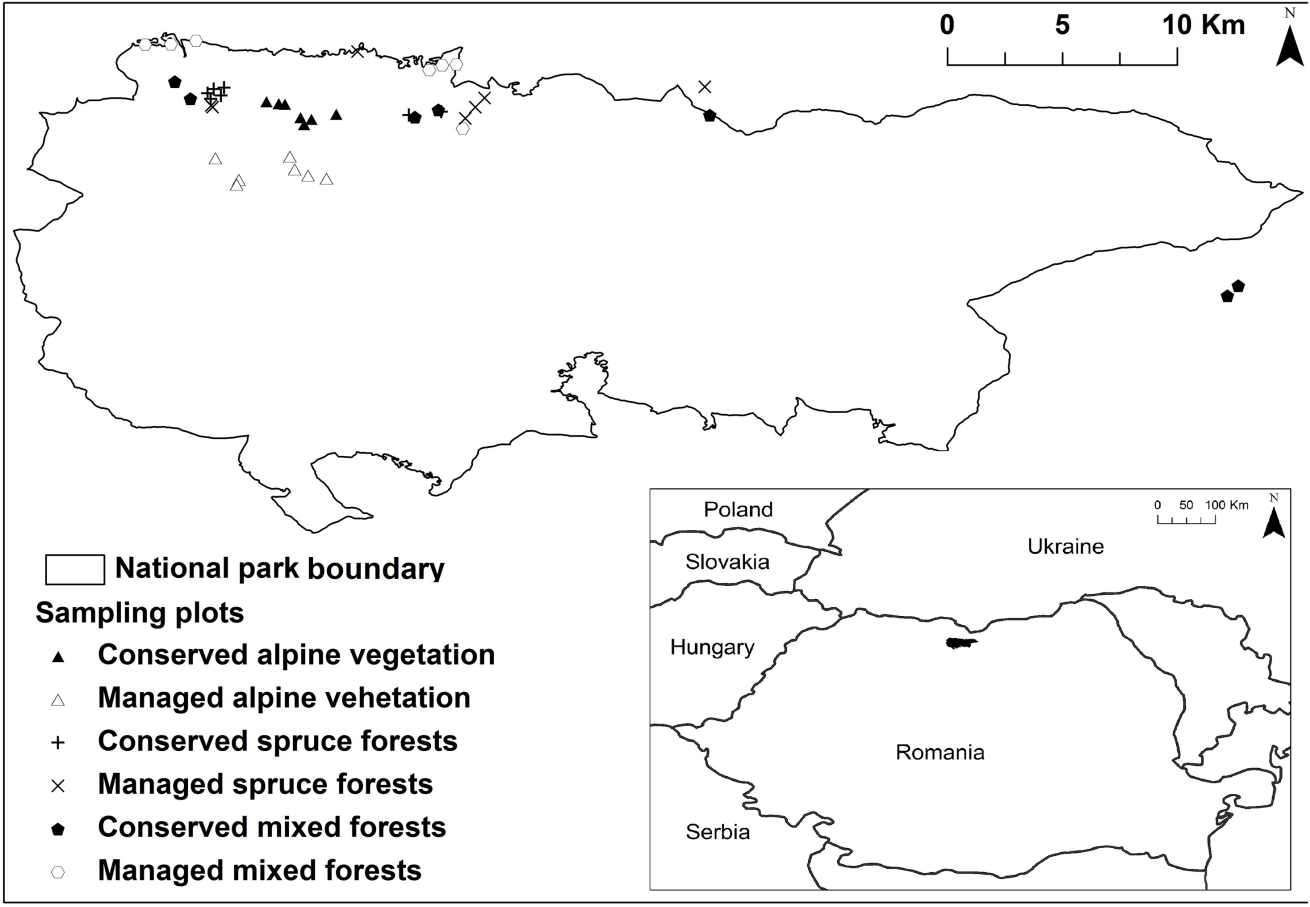
Location of the Rodnei Mountains National Park Romania, with the national park boundary, together with the sampling plots, represented by different symbols, according to the different categories of vegetation type and management.

The study area, which is part of Rodnei Mountain National Park, shelters a high biodiversity, harbouring the highest number of endemic vascular plant species in the South-Eastern Carpathians [15] and numerous glacial relicts and rare species of flora and fauna. It is situated in the northern part of the Eastern Carpathians (Fig 1) and is one of the three UNESCO Biosphere Reserves in Romania, included in the Natura 2000 networks of the European Union [16]. A detailed description of the lichen flora of Rodnei Mountains shows that 442 lichen taxa have been reported in the Rodnei Mountains region [17, 18], including a high number of threatened lichens.

The aim of this study was to analyse how vegetation type, lichen substrates and environmental parameters influence lichen species richness, community composition and lichen ecological traits in the protected area of the Rodnei Mountains National Park. We also studied how management affects lichen community structure. Current land-use in the Rodnei Mountain National Park is either part of the traditional land-use system, e.g. transhumance systems with seasonal cattle grazing in alpine and subalpine meadows, or of the largely uncontrolled timber exploitation in protected areas.

## Materials and Methods

### Study area

The altitude of the National Park varies between 500–2.303 m a.s.l. The moderately continental and slightly North Atlantic climate of the region is influenced by the East-West orientation of the mountain ridges [19]. This mainly impacts the north- and south-exposed slopes, which differ in temperature and precipitation regimes. The mean annual temperatures vary from 6°C at the base of the mountains to -1.5°C on the ridges at around 2300 m a.s.l. Average annual rainfall ranges from 1200 mm in the lower regions to over 1400 mm at higher sites on the mountain sides [20]. The geology mainly consists of crystalline schist substrata [16].

Management history varies among vegetation types. In the alpine meadows it is generally traditional sheep grazing, but at lower altitudes also includes horses and cattle. In the forests the management history is more complex. Until 1948, the management varied according to the owners’ interests and sometimes no management plan was followed. At the beginning of the twentieth century and between the two World Wars, forest exploitation involved mainly clear cutting large areas with no special care taken to ensure forest regeneration. After the Second World War, management planning focused on clear cutting small plots, and replanting gaps with spruce, so that the mixed forests began to lose ground. After 1990, illegal cutting became widespread and was difficult to stop due to the economic and social situation in the area [16]. Given the current largely unregulated management within the borders of the National Park, old-growth forest stands are being transformed into clear-cut areas or managed forests with less structural diversity and habitat heterogeneity, including fewer old and mature trees [4].

### Sampling method

In our study we investigated three vegetation types typical of the National Park, summarised in Table 1, distinguishing two classes of management namely conserved or managed, for each. Our field campaign was authorized by the Rodnei Mts. National Park Administration (entry permit signed by Prof. Gheorghe Coldea, Scientific Director of the National Park) with permission to have access and collect data (including biological material). No permission was required for collecting sites outside the Park borders as they belong to the public domain and are freely accessible.

**Table 1:** Description of environmental variables used in the lichen biodiversity analyses.

Measured variables	Description of categorical variables	No. of samples
Management type	Conserved sites (no human exploitation)	21
	Managed sites (pasturing in the meadows and logging in the forests)	21
Vegetation type	Alpine vegetation (consisting of alpine meadows with bare rocks)	14
	Spruce forests	14
	Mixed forests (composed of beech, spruce, maple, and fir)	14
	Description of continuous variables	Range (min-max)
Altitude	Elevation a.s.l. (in meters)	863–2193
Slope	Mean inclination of the slope (in degrees)	4.958–43.846
Aspect	Northness	-0.99779–0.977885
	Eastness	-0.99982–0.997849

The samples were collected using a replicated design, consisting of seven circular plots of one hectare in size or 56.4 m in radius for each habitat type and corresponding levels of conservation, i.e. 42 plots in total. The minimum distance between the plots was 100 m. In each sampling plot we registered the altitude and the geographic coordinates. The aspect and slope were inferred in GIS from a 30 m cell raster, available from http://www.jspacesystems.or.jp/en_/. Aspect was arcsine transformed, and the exposition expressed as ‘Eastness’ and ‘Northness’ [21].

Six collecting sites in each sampling plot served as the starting point (Table 2) for four lichen relevés (each with a total area of 50x40 cm) according to the method described in Scheidegger *et al*. [22]. One relevé was conducted on each of the following substrates: rocks, soil, bark of living trees and deadwood. All lichens were considered apart from the crustose lichens on the rocks. In the plots where some substrate types were not available or were not colonized by lichens, substitute relevés were made on other substrates hosting lichens, resulting in 24 relevés for each plot.

**Table 2:** Number, azimuth and distance of the six collecting sites in each one-ha sampling plot as measured from the centre of the sampling plot.

Collecting site no.	Azimuth (360° gradation)	Distance from centre (m)
1	0	18.2
2	60	36.4
3	120	18.2
4	180	36.4
5	240	18.2
6	300	36.4

### Data Analysis

We analysed lichens from all substrates taken together, as well as separately from soil, tree and deadwood. Since lichens from rocks were often poorly developed in the forest, we only assessed the saxicolous macrolichens, but did not analyse this group of species separately. Lichen species richness and composition were analysed from relevés on the one-ha plots pooled together.

The variation in lichen species richness, specialist species and red-listed species richness were analysed with Generalized Linear Models (GLMs) using Poisson distribution. The dependent variable was the number of species belonging to the group of interest. Poisson regression provides a model that describes how the mean response of species richness changes as a function of one or more explanatory variables. The “log” link function was used. A previous model selection based on the smallest AICc values using *dredge* function from “MuMIn” package [23] was applied to obtain the best model. The predictor variables were checked for collinearity with the *vif* function from “Car” package [24]. In order to test the model, chi-square “Lack of fit” test and the dispersion were checked for each GLM. As the terricolous species richness GLM showed over dispersion, we excluded them from the results. The deviance for the GLM fits was analysed with *anova*.*glm* function. The p values were adjusted with the “Bonferroni” method in all analyses. In the models where categorical independent variables were significant, we calculated the mean and the standard deviation to see which of the categories had higher values. If more than two categories were compared, the Tukey HSD test was used.

Ecological functional traits included substrate preference as defined by Stofer *et al*. [12]. We considered only the specialist lichens, which are restricted to one substratum. The threatened red-listed species richness included the following IUCN categories: regionally extinct (RE), critically endangered (CR), rare (R), endangered (EN) and vulnerable (VU). Since no official Romanian national lichen Red List exists [17], we compiled a list using data from the literature, based mainly on the proposed Red List of macrolichens from Romania (Bartok & Crișan, personal communication) and the available Red Lists of the surrounding Carpathian countries: Ukraine [25], Slovakia [26] and Poland [27].

Variation in lichen species composition was assessed using Partial Canonical Correspondence Analysis (pCCA) with the chi-square distance. Vegetation type was chosen as a co-variable and all the other measured variables as explanatory variables. The ordination was calculated using the *pCCA* function of the “vegan” package [28]. The model selection for the ordination was made with a stepwise procedure based on the p values, using the *ordistep* function from the “vegan” package. The significance of the explanatory variables was analysed with the ANOVAlike permutation test for Canonical Correspondence Analysis. In order to identify the characteristic species of the two management categories, a subsequent analysis of the species composition was carried out with pCCA using only the management type as the explanatory variable and the vegetation type as the co-variable. Extracting the lichen species scores, those species that were positioned at the ends on the ordination axis were chosen as characteristic for the two types of management: conserved and managed. Only species that occurred in at least four plots were considered in the composition analysis. Statistical analyses were carried out with R version 3.0.2 (2013 The R Foundation for Statistical Computing).

## Results

### Species richness

We found a total of 240 lichen species and one form. Of these, 86 species were substrate specialists (growing on one single substrate) according to the literature [12], 123 species intermediate (growing on two or three different substrates) and 31 species generalists (growing on more than three substrate types). In terms of substrate, we found 102 species on soil, 132 species on trees, 93 species on deadwood and 31 species on rocks. The GLM model of all lichen species showed that the management and vegetation type significantly influenced species richness. Conserved sites had higher mean species richness than managed sites (Table 3). On average, hectare plots in conserved sites harboured 13 species more than managed sites. Comparing the species richness of the vegetation types, mixed forests had the highest species richness per plot, with a mean of 35.6, and the lowest richness was found in the spruce forests, with a mean of 23.5 species per plot.

**Table 3:** Generalized linear models (GLMs) of lichen species richness in relation to the environmental variables measured. The significant variables are in bold. The mean number and the standard deviation (SD) of species richness per plot for each category of significant factor are displayed in the two columns on the right. When all lichens were assessed, the Tukey HSD test showed significant differences according to vegetation type only between the species richness of mixed and spruce forests.

Species richness Model	adjR^2	Dispersion	Df	Deviance	Pr(>Chi)	Species richness	Mean ± (SD)
*All lichens*	0.922	1.068				Conserved sites	35.8 ± (7.2)
**Management type**			**1**	**64.623**	**<0.001.**	Managed sites	22.5 ± (7.4)
**Vegetation type**			**2**	**35.685**	**<0.001.**	Alpine vegetation	28.28 ± (7.22)
Management type: Vegetation type			2	7.147	0.084	Mixed forests	35.6 ± (8.71)
						Spruce forests	23.5 ± (9.9)
*Lichens on trees*	0.98	1.02				Conserved sites	21 ± (9.51)
**Management type**			**1**	**48.346**	**<0.001.**	Managed sites	10.6 ± (5.1)
**Vegetation type**			**1**	**61.408**	**<0.001.**	Mixed forests	21.8 ± (9.07)
						Spruce forests	10 ± (4.22)
*Lichens on deadwood*	0.393	0.836				Mixed forests	13.1 ± (3.63)
Altitude			1	0.0716	1.000	Spruce forests	10.2 ± (3.38)
Eastness			1	2.2489	0.535		
Slope			1	2.4814	0.461		
**Vegetation type**			**1**	**9.1276**	**0.010**		

The findings for lichen species richness on trees were comparable with those for total lichen species richness, but the effect of the management type was even stronger. The species richness in managed forest plots was only 50% of that in conserved plots (Table 3), and that in mixed forests was more than twice as high as that in spruce forests (Table 3). For lichen species on deadwood, only the vegetation type had a significant effect on species richness, with, as expected, higher species richness in mixed forests (Table 3).

### Species composition

Considering all substrate types together, 97 species were found on at least four plots and were therefore used in the composition analysis. Considering the substrate types separately, 40 species that fulfilled the condition of a minimum of four occurrences at the plot level were found on soil, 38 on trees, and 24 on deadwood.

The full model of CCA ordination, using all the independent variables measured as explanatory variables, showed that vegetation type was a strong factor in differentiating lichen community composition in all our analyses. To determine the effects of the other environmental variables measured, we chose the pCCA ordination, using vegetation type as a co-variable (Table 4). The lichen species composition from all substrates taken together was influenced by habitat management and Northness.

**Table 4:** Variation in lichen species composition in relation to the environmental variables, measured with ANOVAlike permutation test.

Data	Environmental variables	DF	F	N. Perm	Pr (>F)
*All lichens*	**Northness**	**1**	**1.6125**	**99**	**0.03**
	**Management type**	**1**	**2.4765**	**99**	**0.01**
	Condition (Vegetation type)	2			
*Lichens on soil*	**Management type**	**1**	**2.4033**	**99**	**0.01**
	Condition (Vegetation type)	2			
*Lichens on trees*	Slope	1	1.4837	999	0.085
	**Altitude**	**1**	**1.6567**	**199**	**0.04**
	**Northness**	**1**	**2.068**	**99**	**0.01**
	**Management type**	**1**	**2.7487**	**99**	**0.01**
	Condition (Vegetation type)	1			
*Lichens on deadwood*	Northness	1	1.8623	399	0.0575
	**Altitude**	**1**	**1.8964**	**99**	**0.03**
	Condition (Vegetation type)	1			

Management type also significantly influenced lichen communities growing on soil, whereas the other explanatory variables did not. The most characteristic species for conserved communities included *Alectoria ochroleuca*, *Arthrorhaphis citrinella*, *Cetraria ericetorum*, *Cladonia gracilis*, *Cladonia subcervicornis* and *Lepraria nivalis*. In the managed sites, the most characteristic species for the lichen communities from soil were: *Cladonia bellidiflora*, *Cladonia cornuta*, *Cladonia maxima*, *Cladonia uncialis* and *Placynthiella icmalea*.

The composition of lichen species from trees was significantly influenced by management type, altitude and Northness. The community differentiation of lichens from trees between conserved and managed sites was greater than it was for all lichens, based on the F values of the ANOVAlike permutation test (Table 4). The characteristic species of lichen communities from trees negatively affected by management included: *Chaenotheca chrysocephala*, *Evernia prunastri*, *Graphis scripta*, *Lecanora argentata*, *Lecanora intumescens*, *Parmeliopsis ambigua*, *Pertusaria leioplaca* and *Usnea filipendula*. In the managed sites, the representative species of the lichen communities were: *Dimerella pineti*, *Graphis pulverulenta*, *Lecanora strobilina*, *Micarea prasina*, *Mycobilimbia epixanthoides*, *Scoliciosporum chlorococcum* and *Scoliciosporum sarothamni*. The lichen species composition from deadwood substrate varied only with altitude (Table 4).

### Substrate specialist lichens

Overall we found 86 substrate specialist lichen species (listed in S1 Appendix), namely 47 epiphytic, 5 lignicolous, 22 terricolous and 12 saxicolous. The GLMs of the substrate specialist lichens were calculated only for all lichens, terricolous lichens and epiphytic lichens.

The species richness of the substrate specialist lichens was influenced by the management type, vegetation type and their interaction. The conserved habitats hosted a larger number of substrate specialist lichens. Regarding the vegetation type, the species richness in spruce forests was significantly lower than in mixed forests and alpine vegetation (Table 5). Terricolous substrate specialists varied significantly in species richness in the three vegetation types and in the interaction of management with vegetation type. The alpine vegetation had a mean of 7.07 species, which is far more than the two forest types, with 0.42 species in the spruce forests and 0.07 in the mixed forests. The species richness of epiphytic lichens was influenced by both management, with more species in the conserved sites, and by vegetation type, with more species in the mixed forests (Table 5).

**Table 5:** Generalized linear models (GLMs) of substrate specialist lichen species richness in relation to the environmental variables measured. The significant variables are in bold. The mean number and the standard deviation (SD) of specialist lichen species richness per plot, for each category of significant factors, are displayed in the two columns on the right. When all lichens were assessed according to vegetation type, the Tukey HSD test showed significant differences between the specialist richness of lichen species in spruce forests and that of both alpine vegetation and mixed forests. For terricolous lichens, the specialist lichen species richness of alpine vegetation was significantly different from that of both spruce forests and mixed forests.

Specialist lichens sp. richness model	adjR^2	Dispersion	Df	Deviance	Pr(>Chi)	Species richness	Mean ± (SD)
*All lichens*	0.95	0.76				Conserved sites	9.52 ± (4.19)
**Management type**			**1**	**28.62**	**<0.01.**	Managed sites	5.09 ± (4.42)
**Vegetation type**			**2**	**80.88**	**<0.01.**	Alpine vegetation	11.14 ± (2.87)
**Management type: Vegetation type**			**2**	**17.70**	**0.014**	Spruce forests	2.64 ± (2.53)
						Mixed forests	8.14 ± (4.27)
*Terricolous lichens*	0.98	0.6				Alpine vegetation	7.07 ± (2)
Management type			1	0.945	0.99	Spruce forests	0.42± (0.85)
**Vegetation type**			**2**	**1.755.592**	**<0.01.**	Mixed forests	0.07 ± (0.26)
**Management type: Vegetation type**			**2**	**9.012**	**0.033**		
*Epiphytic lichens*	0.95	0.57				Conserved sites	6.14 ± (4.67)
**Management type**			**1**	**27.409**	**<0.01.**	Managed sites	2.35 ± (2.2)
**Vegetation type**			**1**	**60.411**	**<0.01.**	Spruce forests	1.42± (1.22)
Northness			1	0.598	1.000	Mixed forests	7.07 ± (3.97)

### Red-listed species

Our species list included 99 red-listed lichens (given in S2 Appendix). These are mainly rare species, of which 30 were found growing on soil, 58 on trees and 27 on deadwood.

When all substrates were analysed together, the species richness of the red-listed lichens was significantly lower in managed plots, with a 50% reduction in managed compared to conserved plots (Table 6). A comparison of the three vegetation types revealed that mixed forests had a significantly higher number of red-listed species than spruce forests. The highest number of red-listed species was found in the conserved mixed forests, with a mean of 16.9 species, while the lowest number was found in managed spruce forests, with a mean of 2.1 species (data not shown). Terricolous red-listed lichen richness was influenced only by vegetation type. The number of red-listed species in alpine vegetation was significantly higher (7.2) than in the mixed and spruce forests (1 and 0.6, respectively). Red-listed epiphytic lichen richness was influenced by forest management and vegetation type, with a strong discriminating response. Conserved sites harboured over 5 times more red-listed species than managed sites (Table 6). The difference between the two forest types is also high, as can be seen in Table 6.

**Table 6:** Generalized linear models (GLMs) of red-listed (RL) lichen species richness in relation to the environmental variables measured. The significant variables are in bold. The mean number and the standard deviation (SD) of RL lichen species richness per plot, for each category of significant factors, are displayed in the two columns on the right. In the case of vegetation type when all lichens were assessed, the Tukey HSD test showed significant differences only between the RL species richness of mixed and spruce forests. For terricolous lichens, the RL lichen species richness of alpine vegetation differed significantly from that of both spruce and mixed forests.

RL sp. richness model	adjR^2	Dispersion	Df	Deviance	Pr(>Chi)	Species richness	Mean ± (SD)
*All lichens*	0.92	1.00				**Conserved sites**	**11.47 ± (5.7)**
**Management type**			**1**	**61.163**	**<0.01.**	**Managed sites**	**4.71 ± (2.57)**
**Vegetation type**			**2**	**40.152**	**<0.01.**	Alpine vegetation	8.85 ± (3.37)
**Management type: Vegetation type**			**2**	**8.544**	**0.014**	**Spruce forests**	**4.5 ± (3.46)**
						**Mixed forests**	**10.93 ± (7.14)**
*Terricolous lichens*						**Alpine vegetation**	**7.21 ± (2.72)**
**Vegetation type**	0.961	1.544	**2**	**122.724**	**<0.01.**	Spruce forests	0.64 ± (1.15)
Management type			1	4.675	0.092	Mixed forests	1 ± (1.47)
Management type: Vegetation type			2	7.08	0.087		
*Epiphytic lichens*	0.985	1.02				**Conserved sites**	**8.14 ± (6.3)**
**Vegetation type**			**1**	**50.238**	**<0.01.**	**Managed sites**	**1.57 ± (1.01)**
**Management type**			**1**	**68.156**	**<0.01.**	**Spruce forests**	**2 ± (1.96)**
						**Mixed forests**	**7.71 ± (6.5)**

## Discussion

### Lichen species richness and composition

Although management favours some species, this gain by no means compensates for the much higher loss it causes. Previous studies have also reported a decrease in lichen species richness related to management intensity [12, 13, 29, 30].

The mixed forests in our study area contain the greatest number of lichen species, and the current management system with selective cutting is a major source of diversity loss in these forests. Selective cutting decreases forest structural diversity [31, 32] which is an important factor for lichens, especially epiphytic ones, through various mechanisms. Studies on epiphytic species richness from primeval forest [33] or old growth forests [34] have shown that forest structural diversity with considerable variation in the age of trees and degree of canopy closure is important for the richness of these lichen species. Epiphytic lichens select their substrate according to the bark properties i.e. corrugation, pH, moisture-holding capacity and nutrient status of the bark [35, 36], which varies with the age of the trees. The distribution of forest lichen species is also influenced by the light regime [37], which relates to canopy closure.

Harvesting mature and old trees mainly affects lichen species that depend on keystone structures [38] found only in old trees, such as bark crevices or soft bark with a high water-storage capacity. They may be found in old beech trees or rain-protected, slightly overhanging trunks of trees with an asymmetric canopy structure [39]. Species within the study area that depend on old trees and that appear to be significantly affected by management include: *Arthonia vinosa*, *Chaenotheca brachypoda*, *Heterodermia speciosa*, *Hypogymnia vittata*, *Lecanora cinereofusca*, *Lobaria pulmonaria*, *Loxospora cismonica*, *Megalospora tuberculosa*, *Menegazzia terebrata*, *Thelotrema lepadinum*, *Usnea florida*, and *Usnea fulvoreagens* [40, 41].

The other forest management type, clear cutting, characteristically in the spruce forests investigated, temporarily removes the habitat of epiphytic lichen substrates even if it is applied to relatively small patches. During subsequent forest growth, the even-aged stands vary very little in their bark substrate types, and light availability is limited for several decades. These drastic changes in environmental conditions target the epiphytic lichens. Their mean species number on trees in the one-hectare plots decreased by 48% after clear-cutting started in 1958 [16].

A recent study detected no differences in species richness between unmanaged and managed sites [42] in formerly managed forests in Germany. The unmanaged forest stands they surveyed, however, had not yet reached the level of structural diversity characteristic of old-growth forests and thus still showed signs of former management. This situation is rather different from that of the forests in our study.

Comparing the response of lichen species richness on trees and deadwood in mixed and spruce-dominated forests, only lichen species from trees are highly sensitive to forest management in previously unmanaged, natural forests (Table 3). Species richness on deadwood was not affected by forest management. Therefore, we presume that stumps are important for species richness in the managed forests, as has frequently been claimed for lignicolous lichens [43]. Another reason could be that stumps in managed forests can act as short-term refuges for species which can grow not only on living trees but also live on deadwood. For example, in Fennoscandia and the Pacific Northwest of North America, Spribille *et al*. [44] found that 43% of the lichens growing on trees there can also grow on deadwood.

Management had negative effects not only on species richness but also on the community composition, which may be significantly changed by management.

The lichen communities on trees proved to be very sensitive to the changes brought about by forest management. They confirmed the high indicator properties of communities in relation to the intensity of the management described in previous studies [13, 45, 46]. The environmental variables represented by altitude and Northness, which are substitutes for climatic conditions in terms of temperature gradients [47], precipitation [20] and light availability, also contributed to variations in the community composition. When these environmental variables were exclude from our constrained ordination (pCCA), the characteristic species of the two management types helped us visualise important processes taking place in the forests studied. The lichen communities from conserved sites mainly consist of common forest species. A high level of richness of Caliciaceae family is considered to indicate ancient forests [36], but only *Chaenotheca chrysocephala* was frequent in our conserved forests, and this species is rather common.

The frequency of some species, such as *Usnea filipendula*, which are commonly found across Europe, declines with forest exploitation both in Europe generally [41] and in our study area. Our conserved forests also contain rare or overlooked characteristic species, such as *Lecanora argentata* and *L*. *intumescens* [48]. It is, however, surprising that the indicator species characteristic of old-growth forests are not represented better in our lichen community assessment in the conserved forest, given the high number of such species found in our research area [17]. In the community composition analysis, we filtered out those species that occurred in less than four plots and excluded them from our assessment. The analysis showed that the lichen species that are most characteristic of old-growth forest were not very frequent in our study area. We assume the reason for this lack of frequency is probably that the managed forests fragment the conserved forests so that the dispersal of old-growth forest species is limited by ecological barriers.

Characteristic lichen communities on trees in managed forests are dominated by common early successional lichen species, which are widely distributed, [41]. The ecological preferences of the lichen species *Dimerella pineti*, *Micarea prasina* and *Scoliciosporum chlorococcum* show that the managed forests we investigated are predominantly shaded. The limited light represents a filter for a large number of lichen species.

The composition variability of lichens growing on soil requires careful interpretation because the alpine vegetation category is not properly distributed across altitude. The conserved sites are at higher altitudes than the managed sites (S3 Appendix), which could induce variability in the lichen composition. Sheep grazing may affect the composition of the lichen community positively or negatively, as some new species may be added while other more sensitive species are eliminated through trampling [49]. The trampling effect is even stronger with grazing cattle [50], as is the case in our sample plots at lower altitudes. Moreover, plants respond to grazing in different ways: with some species the abundance increases because they have developed a resistance to this type of disturbance [51]. Thus the competition with the plants is stronger in the managed alpine meadows for the terricolous lichens. The characteristic lichens found in the conserved meadows are typical alpine species, some of them rare (e.g. *Alectoria ochroleuca*, *Cetraria ericetorum*, and *Cladonia subcervicornis*). Their growth could be restricted by the stronger competition from the vascular plants at lower altitudes [52].

The managed alpine site communities include characteristic lichen species with wider distribution ranges (i.e. *Cladonia bellidiflora* and *C*. *cornuta*), or ruderal species such as *Placynthiella icmalea*, which has adapted to a wide range of substrates and is known as a primary coloniser [48].

The lichen communities from soil substrates in the forest did not appear to differ in the managed and unmanaged plots, but unlike in alpine vegetation, soil is not among the main substrates for lichens in forests due to their low competition abilities [53].

### Management affects mainly red-listed and substrate specialist lichen species

Our results showed that management reduced the mean level of threatened species richness by 59%, taking all lichen species into consideration, and by 81% if only epiphytic lichens were considered. This suggests that, among all lichen species, threatened lichens are the most affected by forest exploitation, with all the subsequent changes it brings about. This is in accordance with previous findings [10, 54, 55]. The substantial decline in threatened lichen species taking place in our study area is alarming considering that this was observed in an overall protected area, where any tree cutting is largely an unregulated and unplanned action. The study region was declared a biosphere reserve in 1979 and was progressively enlarged up to 2003, to maintain the regional biodiversity [16] in an otherwise intensively managed forest landscape. This decline is the result of past forest management that spanned several decades, but also the one practiced nowadays.

Some specialised lichens have strong substrate preferences and grow only on one substrate type. At the opposite extreme are the generalist lichens, which grow on at least three types of substrates [12]. In a biodiversity assessment study, Stofer *et al*. [12] used functional traits of lichens to describe the significant increase in species richness of generalist lichens along an anthropization gradient. Unlike in our study, they found no clear distribution pattern for the specialised lichens in the major ecoregions of continental Europe. In our case, the responses of substrate-specialist lichens indicated that the number of all lichens, as well as of terricolous and epiphytic lichens, was significantly smaller in managed sites than in conserved sites. This clearly implies that habitat management affects the regional species pool of lichens.

We found several specialised epiphytic lichens on deadwood substrate in the conserved forests, namely *Candelariella reflexa*, *Graphis pulverulenta*, *Lecidella subviridis*, *Opegrapha viridis*, *Parmelia submontana* and *Porina aenea*. This suggests that there is continuity between living tree and deadwood substrates, and the deadwood decadal stages in these forests are very complex.

### Conservation of lichen diversity

Although we found that management decreased overall lichen diversity, effective conservation measures differ between forest and grassland ecosystems. For terricolous lichens from the alpine meadows grazing must be controlled in intensively grazed pastures to allow the typical lichen communities to persist [56]. In some habitats, grazing is important to sustain higher lichen diversity and save some species from local extinction [57]. In our study region, the diversity of terricolous lichen was changed, even by low intensity grazing. In order to conserve the typical alpine lichen flora, only grazing by wild animals should be allowed, and pasturing by domestic animals should be restricted. This is the case in the strictly protected alpine meadows in the Rodnei Mountains National Park, where the lichen diversity is high thanks to conservation.

Conservation measures for forest epiphytic and lignicolous lichens depend on the forest management type [58]. For managed forests, the most important recommendation is to extend the rotation periods of forest exploitations extended [59–61] and of selective cutting in order to create structurally heterogeneous landscapes with a range of different habitats [62]. However, in most European forest types, lichen diversity, especially that of threatened and red-listed species, depends on the presence of old trees [34, 42, 55, 63], because they provide keystone structures for up to 70% of rare and threatened lichen species [39, 40, 64]. Old-growth forests therefore play an important role as lichen sanctuaries and refuges because they maintain viable lichen populations and serve as sources of lichen propagules for neighbouring managed habitats [65]. Old-growth forests thus merit a high conservation priority and should be strictly protected.

Habitat size and heterogeneity influence lichen diversity [10, 66]. Since species drift is likely to increase in small and isolated fragments of habitat due to the dispersal limitation of the species set [67], the primary focus of conservation strategies for lichens should be, according to Scheidegger and Werth [65], the maintenance of habitat quality, connectivity and size. This focus fits in well with the objectives of the protected area in the National Park, which is also a UNESCO Biosphere reserve.

At present, the strictly protected areas (category I IUCN) in the National Park include mainly alpine meadows, shrubs and spruce forest vegetation belts, while mixed forests are not well represented [16]. The vegetation is distributed in 300–500 m belts along the altitudinal gradient, but mixed forests are only present in the altitudinal interval of 650–1100 m [19]. Moreover, the zonation of protection levels in the National Park includes peripheral areas where forest exploitation is allowed, which often affects mixed forests as they are mostly confined to lower altitudes.

## Conclusions

Lichen diversity is greater in conserved than in managed sites. Management decisions are thus very important as they influence the human impact on the vegetation types (i.e., alpine meadows and the forests) of this UNESCO Biosphere Reserve. Careful decisions in the management of natural resources should be taken not only within the borders of protected areas, but overall, in order to diminish the diversity loss as much as possible.

The protected area investigated includes forests that, although under protection status in present, have a history with intensive exploitation [16], and have low lichen diversity. Conversely, some of the currently well-preserved mixed forests with high lichen diversity are outside its borders (Fig 1) and urgently require protection status. We therefore strongly recommend maintaining the high level of protection within the current boundaries of the protected areas and re-evaluating the boundaries of the national park. The re-evaluation is necessary especially for the category I IUCN protection zone, to ensure a sufficiently large area of representative vegetation types of the National Park.

## Supporting Information

S1 Appendix Specialist lichen species ordered according to substrate type. (DOCX)

S2 Appendix Threatened red-listed lichens considered in our analysis, with the proposed Red List of macrolichens from Romania (RL Ro), the Red List of Ukraine (RL Uk), Poland (RL Pl) and Slovakia (RL Sl). The following IUCN categories are abbreviated in the table: regionally extinct (RE), critically endangered (CR), (R) rare, endangered (EN), vulnerable (VU).
(DOCX)

S3 Appendix Mean values and standard deviation (SD) of the continuous independent variables for each category of sampled site. (DOCX)
