# Supplementary material for: Effects of Management on Lichen Species Richness, Ecological Traits and Community Structure in the Rodnei Mountains National Park (Romania)
Source: PLoS One. 2015 Dec 30;10(12):e0145808. doi: 10.1371/journal.pone.0145808 (PMC4696781; doi:10.1371/journal.pone.0145808)
Supplement: S1 Appendix — (DOCX) [file pone.0145808.s001.docx]

**S1 Appendix. Specialist lichen species ordered according to substrate type.**

| Epiphytic lichens | *Thelotrema lepadinum* |
| --- | --- |
| *Acrocordia gemmata* | *Usnea chaetophora* |
| *Anisomeridium biforme* | *Usnea subfloridana* |
| *Arthonia caesia* | Lignicolous lichens |
| *Arthonia didyma* | *Micarea misella* |
| *Arthonia elegans* | *Peltigera horizontalis* |
| *Arthonia lapidicola* | *Trapeliopsis viridescens* |
| *Arthonia punctiformis* | *Xylographa parallela* |
| *Arthonia radiata* | *Xylographa vitiligo* |
| *Arthonia vinosa* | Terricolous lichens |
| *Biatora efflorescens* | *Baeomyces placophyllus* |
| *Biatora globulosa* | *Bryoria chalybeiformis* |
| *Bryoria capillaris* | *Bryoria implexa* |
| *Bryoria lanestris* | *Cetraria ericetorum* |
| *Buellia disciformis* | *Cetraria islandica* |
| *Buellia erubescens* | *Cladonia arbuscula* |
| *Calicium cf. salicinum* | *Cladonia bellidiflora* |
| *Candelariella reflexa* | *Cladonia callosa* |
| *Chaenotheca brachypoda* | *Cladonia cervicornis* |
| *Evernia divaricata* | *Cladonia macroceras* |
| *Fuscidea arboricola* | *Cladonia maxima* |
| *Fuscidea pusilla* | *Cladonia rangiformis* |
| *Graphis pulverulenta* | *Cladonia symphycarpia* |
| *Graphis scripta* | *Dibaeis baeomyces* |
| *Japewia subaurifera* | *Flavocetraria cucullata* |
| *Lecanora albella* | *Lecidella elaeochroma f. soralifera* |
| *Lecanora argentata* | *Micarea lignaria* |
| *Lecanora cf. hybocarpa* | *Micarea turfosa* |
| *Lecanora cinereofusca* | *Peltigera lepidophora* |
| *Lecanora intumescens* | *Pycnothelia papillaria* |
| *Lecanora subrugosa* | *Thamnolia vermicularis* |
| *Lecidea leprarioides* | *Trapeliopsis gelatinosa* |
| *Lecidella subviridis* | Saxicolous lichens |
| *Loxospora cismonica* | *Brodoa intestiniformis* |
| *Melanohalea elegantula* | *Cornicularia normoerica* |
| *Opegrapha niveoatra* | *Melanelia stygia* |
| *Opegrapha viridis* | *Pseudephebe pubescens* |
| *Opegrapha vulgata* | *Sphaerophorus fragilis* |
| *Parmelia submontana* | *Stereocaulon alpinum* |
| *Pertusaria leioplaca* | *Umbilicaria crustulosa* |
| *Porina aenea* | *Umbilicaria cylindrica* |
| *Pyrenula nitida* | *Umbilicaria deusta* |
| *Pyrenula nitidella* | *Umbilicaria polyphylla* |
| *Ropalospora viridis* | *Umbilicaria vellea* |
| *Scoliciosporum sarothamni* |  |
