# Supplementary material for: Effects of Management on Lichen Species Richness, Ecological Traits and Community Structure in the Rodnei Mountains National Park (Romania)
Source: PLoS One. 2015 Dec 30;10(12):e0145808. doi: 10.1371/journal.pone.0145808 (PMC4696781; doi:10.1371/journal.pone.0145808)
Supplement: S2 Appendix — The following IUCN categories are abbreviated in the table: regionally extinct (RE), critically endangered (CR), (R) rare, endangered (EN), vulnerable (VU). (DOCX) [file pone.0145808.s002.docx]

**S2 Appendix. Threatened red-listed lichens considered in our analysis, with the proposed Red List of macrolichens from Romania (RL Ro), the Red List of Ukraine (RL Uk), Poland (RL Pl) and Slovakia (RL Sl). The following IUCN categories are abbreviated in the table: regionally extinct (RE), critically endangered (CR), (R) rare, endangered (EN), vulnerable (VU).**

| Lichen taxa | RL Ro | RL Uk | RL Pl | RL Sl |
| --- | --- | --- | --- | --- |
| *Absconditella lignicola* Vězda & Pišút |  |  | VU |  |
| *Anisomeridium biforme* (Borrer) R.C. Harris |  |  | VU | RE |
| *Arthonia didyma* Körb. |  |  | EN | CR |
| *Arthonia elegans* (Ach.) Almq*.* |  |  | RE |  |
| *Arthonia punctiformis* Ach. |  |  | EN | CR |
| *Arthonia radiata* (Pers.) Ach. |  |  |  | CR |
| *Arthonia vinosa* Leight. |  |  |  | CR |
| *Arthrorhaphis citrinella* (Ach.) Poelt |  |  | VU |  |
| *Bacidia rubella* (Hoffm.) A. Massal. |  |  | VU | VU |
| *Baeomyces placophyllus* Ach. |  |  | CR |  |
| *Bellemerea cinereorufescens* (Ach.) Clauzade & Cl. Roux |  |  | CR |  |
| *Biatora efflorescens* (Hedl.) Räsänen |  |  | VU | VU |
| *Brodoa intestiniformis* (Vill.) Goward |  |  | VU |  |
| *Bryoria capillaris* (Ach.) Brodo & D. Hawksw. |  |  | CR | CR |
| *Bryoria chalybeiformis* (L.) Brodo & D. Hawksw. |  |  | CR |  |
| *Bryoria implexa* (Hoffm.) Brodo & D. Hawksw. |  |  | CR | CR |
| *Bryoria lanestris* (Ach.) Brodo & D. Hawksw. | VU |  | RE | CR |
| *Buellia disciformis* (Fr.) Mudd |  |  | VU |  |
| *Buellia erubescens* Arnold |  |  | CR |  |
| *Calicium salicinum* Pers. |  |  | VU | EN |
| *Cetraria ericetorum* Opiz |  |  |  | VU |
| *Cetraria islandica* (L.) Ach. | VU |  | VU | VU |
| *Cetrelia olivetorum* (Nyl.) W.L. Culb. & C.F. Culb. |  |  | EN |  |
| *Chaenotheca brachypoda* (Ach.) Tibell |  |  | EN | CR |
| *Chaenotheca brunneola* (Ach.) Müll. Arg. |  |  | EN | CR |
| *Chaenotheca chrysocephala* (Ach.) Th. Fr. |  |  |  | VU |
| *Chaenotheca gracilenta* (Ach.) Mattsson & Middelb. |  |  |  | CR |
| *Chaenotheca trichialis* (Ach.) Hellb. |  |  |  | CR |
| *Chrysothrix candelaris* (L.) J.R. Laundon |  |  | CR |  |
| *Cladonia bellidiflora* (Ach.) Schaer*.* |  |  | EN |  |
| *Cladonia caespiticia* (Pers.) Flörke |  |  | EN |  |
| *Cladonia carneola* (Fr.) Fr. |  |  | CR | EN |
| *Cladonia cornuta* (L.) Hoffm. |  |  |  | VU |
| *Cladonia incrassata* Flörke | EN |  | EN | RE |
| *Cladonia macroceras* (Delise) Ahti |  |  | VU |  |
| *Cladonia macrophylla* (Schaer.) Stenh. | VU |  | CR | EN |
| *Cladonia parasitica* (Hoffm.) Hoffm. |  |  | EN |  |
| *Cladonia subcervicornis* (Vain.) Kernst. |  |  | CR |  |
| *Cladonia sulphurina* (Michx.) Fr. | CR |  |  | EN |
| *Collema flaccidum* (Ach.) Ach. |  |  | EN | EN |
| *Cornicularia normoerica* (Gunnerus) Du Rietz |  |  | VU |  |
| *Evernia divaricata* (L.) Ach. |  |  | CR | CR |
| *Evernia prunastri* (L.) Ach. |  |  |  | EN |
| *Flavocetraria cucullata* (Bellardi) Kärnefelt & A. Thell |  |  | VU |  |
| *Graphis scripta* (L.) Ach. |  |  |  | EN |
| *Heterodermia speciosa* (Wulfen) Trevis. |  | R | CR | CR |
| *Hypogymnia farinacea* Zopf |  |  | VU | VU |
| *Hypogymnia vittata* (Ach.) Parrique |  |  | CR | VU |
| *Icmadophila ericetorum* (L.) Zahlbr. |  |  | EN | CR |
| *Lecanactis abietina* (Ehrh. ex Ach.) Körb. |  |  |  | CR |
| *Lecanora albella* (Pers.) Ach. |  |  | EN | CR |
| *Lecanora cinereofusca* H. Magn. |  |  |  | CR |
| *Lecanora intumescens* (Rebent.) Rabenh. |  |  | EN | EN |
| *Lecidoma demissum* (Rutstr.) Gotth. Schneid. & Hertel |  |  | EN |  |
| *Lichenomphalia hudsoniana* (H.S. Jenn.) Redhead *et al.* |  | R |  | VU |
| *Lobaria pulmonaria* (L.) Hoffm. | VU | VU | EN | CR |
| *Loxospora cismonica* (Beltr.) Hafellner |  |  | CR | RE |
| *Loxospora elatina* (Ach.) A. Massal. |  |  | EN |  |
| *Melanelixia subaurifera* (Nyl.) O. Blanco *et al.* | VU |  |  |  |
| *Melanohalea elegantula* (Zahlbr.) O. Blanco *et al.* |  | R | VU |  |
| *Menegazzia terebrata* (Hoffm.) A. Massal. |  |  | CR | CR |
| *Mycoblastus sanguinarius* (L.) Norman |  |  | VU | CR |
| *Nephroma parile* (Ach.) Ach. |  | VU | CR | CR |
| *Ochrolechia androgyna* (Hoffm.) Arnold |  |  | VU |  |
| *Opegrapha varia* Pers. |  |  |  | VU |
| *Opegrapha viridis* Pers. |  |  | VU | VU |
| *Opegrapha vulgata* (Ach.) Ach. |  |  | VU | VU |
| *Parmelia omphalodes* (L.) Ach. |  |  | EN |  |
| *Parmelia submontana* Nádv. |  |  | VU | CR |
| *Parmeliopsis hyperopta* (Ach.) Vain. |  |  | VU | VU |
| *Peltigera degenii* Gyeln. |  |  | VU | VU |
| *Peltigera horizontalis* (Huds.) Baumg. |  |  | EN |  |
| *Peltigera lepidophora* (Nyl.) Bitter | CR |  | EN | CR |
| *Peltigera praetextata* (Flörke ex Sommerf.) Zopf |  |  | VU |  |
| *Pertusaria coccodes* (Ach.) Nyl. |  |  |  | VU |
| *Pertusaria leioplaca* DC. |  |  |  | CR |
| *Pertusaria pertusa* (L.) Tuck. |  |  | VU |  |
| *Phaeophyscia endophoenicea* (Harm.) Moberg |  |  | EN | CR |
| *Pseudephebe pubescens* (L.) M. Choisy |  |  | EN |  |
| *Pycnothelia papillaria* (Ehrh.) L.M. Dufour |  |  | EN | VU |
| *Pyrenula nitida* (Weigel) Ach. |  |  | VU | EN |
| *Pyrenula nitidella* (Flörke ex Schaer.) Müll. Arg. |  |  | EN | CR |
| *Ramalina canariensis* J. Steiner |  | R |  |  |
| *Ramalina farinacea* (L.) Ach. |  |  | VU | EN |
| *Ramalina fastigiata* (Pers.) Ach. |  |  | EN | EN |
| *Ramalina pollinaria* (Westr.) Ach. |  |  | VU | VU |
| *Sphaerophorus fragilis* (L.) Pers. |  |  | EN | VU |
| *Stereocaulon alpinum* Laurer | VU |  | EN | VU |
| *Thamnolia vermicularis* (Sw.) Schaer. |  | VU |  |  |
| *Thelotrema lepadinum* (Ach.) Ach. |  |  | EN | CR |
| *Tuckermannopsis chlorophylla* (Willd.) Hale | VU |  | VU |  |
| *Umbilicaria crustulosa* (Ach.) Lamy |  |  | EN |  |
| *Umbilicaria vellea* (L.) Ach. |  |  | EN | VU |
| *Usnea diplotypus* Vain. |  |  | RE |  |
| *Usnea filipendula* Stirt. |  |  | VU | CR |
| *Usnea hirta* (L.) Weber exF.H. Wigg. |  |  | VU | VU |
| *Usnea subfloridana* Stirt. |  |  | EN | CR |
| *Xylographa parallela* (Ach.) Fr. |  |  | EN |  |
| *Xylographa vitiligo* (Ach.) J.R. Laundon |  |  |  | EN |
