# Supplementary material for: Effects of Management on Lichen Species Richness, Ecological Traits and Community Structure in the Rodnei Mountains National Park (Romania)
Source: PLoS One. 2015 Dec 30;10(12):e0145808. doi: 10.1371/journal.pone.0145808 (PMC4696781; doi:10.1371/journal.pone.0145808)
Supplement: S3 Appendix — (DOCX) [file pone.0145808.s003.docx]

**S3 Appendix. Mean values and standard deviation (SD) of the continuous independent variables for each category of sampled site.**

|  | Mean + SD | | | |
| --- | --- | --- | --- | --- |
| Categories of sampled sites | Altitude | Slope | N | E |
| Conserved alpine vegetation | 2122,3 (±94) | 26 (±16) | -0,4 (±0,6) | -0,05 (±0,77) |
| Managed alpine vegetation | 1806,3 (±342) | 19,8 (±9) | -0,7 (±0,4) | -0,1 (±0,58) |
| Conserved spruce forests | 1386,1 (±140) | 32,5 (±5) | -0,6 (±0,5) | -0,28 (±0,6) |
| Managed spruce forests | 1188,4(±159) | 26,6 (±8) | 0,6 (±0,6) | -0,2 (±0,5) |
| Conserved mixed forests | 1052,6(±120) | 29,3 (±12) | 0,3 (±0,5) | 0,05 (±0,8) |
| Managed mixed forests | 1017,9 (±155) | 29 (±14) | 0,001 (±0,7) | 0,13 (±0,8) |
